# Rigorous Performance Evaluation of Smartphone GNSS/IMU Sensors for ITS Applications

**DOI:** 10.3390/s16081240

**Published:** 2016-08-05

**Authors:** Vassilis Gikas, Harris Perakis

**Affiliations:** School of Rural and Surveying Engineering, National Technical University of Athens (NTUA), Zographos 15780, Greece; hperakis@central.ntua.gr

**Keywords:** smartphone, GNSS, MEMS IMU, precision, trueness, intelligent transportation system

## Abstract

With the rapid growth in smartphone technologies and improvement in their navigation sensors, an increasing amount of location information is now available, opening the road to the provision of new Intelligent Transportation System (ITS) services. Current smartphone devices embody miniaturized Global Navigation Satellite System (GNSS), Inertial Measurement Unit (IMU) and other sensors capable of providing user position, velocity and attitude. However, it is hard to characterize their actual positioning and navigation performance capabilities due to the disparate sensor and software technologies adopted among manufacturers and the high influence of environmental conditions, and therefore, a unified certification process is missing. This paper presents the analysis results obtained from the assessment of two modern smartphones regarding their positioning accuracy (i.e., precision and trueness) capabilities (i.e., potential and limitations) based on a practical but rigorous methodological approach. Our investigation relies on the results of several vehicle tracking (i.e., cruising and maneuvering) tests realized through comparing smartphone obtained trajectories and kinematic parameters to those derived using a high-end GNSS/IMU system and advanced filtering techniques. Performance testing is undertaken for the HTC One S (Android) and iPhone 5s (iOS). Our findings indicate that the deviation of the smartphone locations from ground truth (trueness) deteriorates by a factor of two in obscured environments compared to those derived in open sky conditions. Moreover, it appears that iPhone 5s produces relatively smaller and less dispersed error values compared to those computed for HTC One S. Also, the navigation solution of the HTC One S appears to adapt faster to changes in environmental conditions, suggesting a somewhat different data filtering approach for the iPhone 5s. Testing the accuracy of the accelerometer and gyroscope sensors for a number of maneuvering (speeding, turning, etc.,) events reveals high consistency between smartphones, whereas the small deviations from ground truth verify their high potential even for critical ITS safety applications.

## 1. Introduction

In recent years, the rapid advancements in the technological capabilities of Personal Data Assistants (PDA) and smartphones together with the continuous decrease in their production costs has led to an enormous increase in the number of users worldwide. The reported percentage of smartphone ownership in emerging and developed countries worldwide has risen from about 21% in 2013 to 37% in 2015, whereas overwhelming majorities in most countries surveyed report owing some form of a mobile handset [1]. Moreover, the multi-modal capabilities of all modern devices has resulted a departure from traditional forms of communication services to new, emerging applications that cover a broad spectrum of information exchange in a dynamic manner. Today, in addition to standard communication facilities and Internet/Bluetooth connection capabilities, contemporary smartphones feature embedded, miniaturized navigation sensors suitable to support a wide range of personal mobility (PM) and navigation applications. These sensors, in addition to Global Navigation Satellite (GNSS) chipsets, involve accelerometers, gyroscopes, barometers, and magnetic field sensors as well as video cameras.

In the transport sector, the geolocation sensors and the software tools currently embedded in modern mobile phones, combined with their broad use worldwide has led to the deployment of various new applications ranging from the provision of transport information services to traffic modeling, driver behavior studies and even more specialized applications. Special interest is placed today in the indoors environment [2,3,4,5,6]; however, this study is confined to the outdoors case. Typical examples in the outdoors environment found in the literature include the development of traffic monitoring and prediction systems [7,8], the setup of route choice models [9], the collection of intersection performance data [10], driver behavior studies [11,12], as well as, traffic flow analyses [13,14]. Other studies deal with more specialized transportation topics, such as carbon emission footprint estimation [15] and evaluation of smartphone inertial sensors for cross-platform mobile applications [16]. More recently, crowdsourced smartphone data have also been used in the context of smart cities for emerging mobility applications, including pedestrian mobility management [17], and models to measure the workload associated with the processes of social internet of vehicles [18].

Nevertheless, while such applications rely mostly on collecting, managing and sharing information between the end-users and/or a processing center, the requirements for increased positioning quality are of lesser importance. Today, the navigation sensors embedded in modern smartphones and the data fusion algorithms they employ can deliver user position information even in areas with relatively poor GNSS signal reception. In the past, many researchers have studied the performance capabilities of mobile phone geolocation sensors used for static and GIS-related applications [19,20,21,22]. Nevertheless, despite the fact that smartphones are very attractive for high precision kinematic applications [23,24], published results assessing their actual and in-depth navigation performance are very rare in the literature [25,26,27].

This paper presents a testing methodology and attempts a performance characterization of the navigation sensors and positioning capabilities of two modern smartphones through extensive field testing in a rigorous and rather holistic way. Our aim is to study the potential and limitations and quantify the actual behavior of the navigation sensors and associated software on the basis of trajectory and kinematic parameters comparisons between those obtained from the smartphones and those derived using a high-end GNSS/IMU navigation system. Specifically, this study contains the field procedures, the analysis and assessment results obtained for a number of a passenger vehicle cruising and maneuvering tests undertaken at various driving and environmental (i.e., open and heavily obscured sky) conditions. Analysis is performed in terms of critical user requirement parameters, specifically positioning accuracy (precision and trueness) and availability.

The remaining of the paper is organized in six sections. The following section offers an overview of smartphone location technologies and the state-of-the-art on the topic, whereas Section 3 provides the theoretical principles underlying the testing procedure adopted in this study. Section 4 contains some base information about the mobile phones and geodetic-grade navigation equipment used in this study, the observation scenarios adopted as well as the sensors setup and raw data acquisition. Data processing and reference trajectory establishment are covered in Section 5. Smartphone data analysis and evaluation of the navigation solution is presented in Section 6. Section 7 offers a critical discussion on the potential and implications using smartphones in various ITS and road applications, while some concluding remarks and a brief outline for future research are presented in Section 8.

## 2. Location Technologies in Smartphones

Modern smartphones can provide user position and navigation information using various sensor technologies that employ variants of principles of operation. At a generic level, location technologies in mobile phones can be classified as Radio Frequency (RF), Global Navigation Satellite System (GNSS) and inertial technologies.

The most prominent RF technology used for positioning in smartphones are Wireless Local Area Networks (WLANs),which rely on access points readily available in many environments. WLANs are based on the IEEE 802.11 standard and do not require line of sight information for position estimation [28]. The most popular approach used for WLAN positioning is based on Receiver Signal Strength Indicator (RSSI) information [29]. The Cell of Origin (CoO) and fingerprinting (FP) are the most commonly used positioning techniques, whereas multilateration techniques based on Time of Arrival (ToA) information are less favored due to the excessive complexity found in the time delay imposed during signal propagation [30]; however, is worth mentioning that recent studies [31] under circumstances claim ranging accuracies of the order of centimeters. Other RF technologies that can be used for positioning in smartphones rely on communication protocols such as Bluetooth [30], which have been designed for short range wireless transfer. Typical communication ranges for WLAN range between 50 m to 100 m, and therefore, WLAN technology can only be used effectively in areas in which a dense network of access points is available.

For applications demanding a higher positioning accuracy standard and a wider effective area of operation, GNSS technology is more suitable. GNSS offers a world-wide coverage, it is independent of local infrastructure, and geolocation can be easily accomplished using the multilateration technique to the signals received from the satellites. However, GNSS performance can be heavily affected by obstructions commonly found in most transportation facilities. Tunnels, tall buildings, tree foliage and surrounding vehicles form typical sources of obstacles that may attenuate, interfere or even completely block the satellite signals resulting in deteriorated positions or the inability to compute a user location [11,32]. Today, Assisted GNSS (A-GNSS) and High Sensitivity GNSS (HS-GNSS) chipsets are employed in modern smartphones. These receivers are coupled with a wireless telecommunication modem using data transmitted via a remote system to speed up and improve navigation calculations [29]. Moreover, they facilitate reception of weak signals through improving their signal to noise ratio. This is accomplished through integration over multiple observation intervals and accepting longer acquisition times [33], resulting in a certain position solution that might be of an adequate quality depending on application technical specifications.

Inertial technology is used in kinematic applications to provide user location information based on acceleration measurements [34]. Although it performs independently of operating environment, the quality of the navigation solution drifts out rapidly with time, and therefore, can be hardly used alone. In smartphones, inertial sensors are setup in the form of a custom Micro Electro Mechanical Sensors (MEMS) IMU. A MEMS IMU consists of three orthogonally arranged accelerometers to sense motion, three gyroscopes to sense angular rates, and potentially, three magnetometers to measure the strength of the Earth’s magnetic field. In practice, the GNSS and IMU units operate as a unique system through implementing some form of Kalman filtering that integrates all observables and motion dynamics to provide an optimal navigation solution [35,36,37]. As the technology in wireless and electronic systems evolves, it is expected that the next generation of smartphone devices will feature more advanced localization systems, capable to integrate information from other sources, such as cameras and Radio Frequency IDentification (RFID) units [29,38].

## 3. Mathematical Foundation and Research Methodology

**Figure 1:**
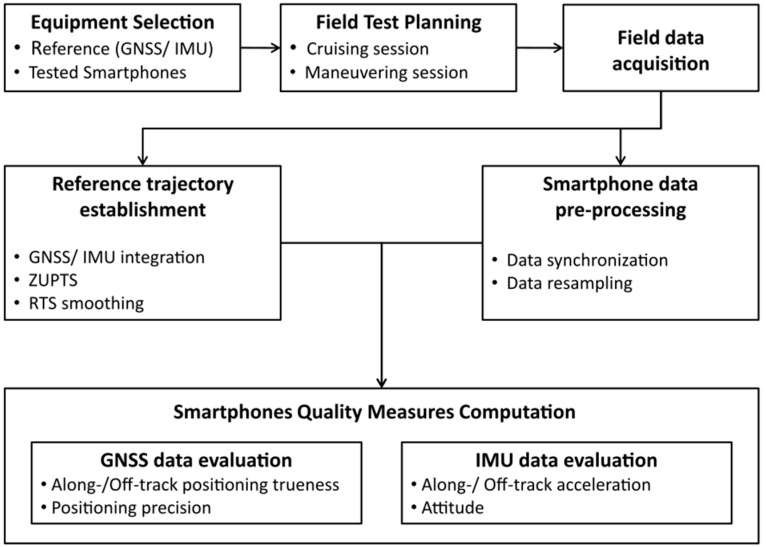
Research methodology flow-chart.

A performance characterization process of any positioning system relies on a number of parameters defined in accordance to the specific needs of a particular application, known as user requirements. Such requirements may relate to positioning, man-machine interface, security and privacy or cost specifications [30]. This study confines in performance characterization of the smartphone navigation sensors regarding their positioning requirements, particularly positioning accuracy realized in terms of precision and trueness. For this purpose a testing methodology was developed and implemented as described in Section 3.2. An overview of the testing methodology procedure is presented in the flow chart of Figure 1.

### 3.1. Positioning Precision and Trueness

In navigation and guidance, positioning accuracy takes the form of precision and trueness. Precision characterizes the performance of a vehicle navigation system that relies solely on its own error estimates and refers to the repeatability or reproducibility of measurements. Instead, trueness of a vehicle trajectory expresses the closeness of agreement between the observed navigation solution to the actual (i.e., true) trajectory [39]. In statistical terms, the “dispersion” of the error probability distribution of a positioning terminal reflects its precision capability, whereas its “mean” indicates the deviation from the true trajectory which is associated to the positioning trueness or reliability of the system. Depending on the case, accuracy measures can be visualized in various ways. Standard representations are perceived in the time and frequency domains, from which the latter usually takes the form of a probability density function or a cumulative probability distribution.

### 3.2. Testing Methodology

**Figure 2:**
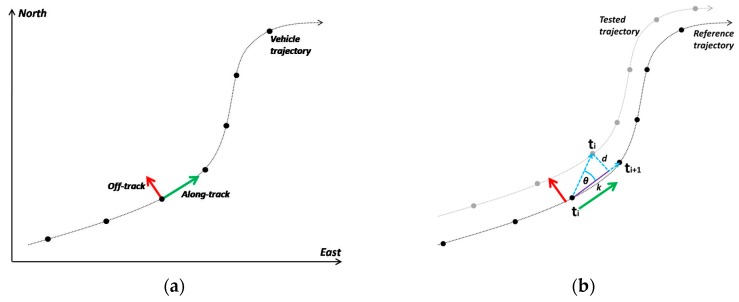
Along-/off-track coordinate system (i.e., body frame) (**a**), and observation geometry involved in positioning trueness definition of a moving vehicle (**b**).

In this study, in order to evaluate the positioning accuracy of smartphone trajectories we turn the originally accuracy values computed in a global coordinate system (e.g., Eastings, Northings) in their along-track and off-track components, so that, they can directly be related to motion (longitudinal and lateral) characteristics (Figure 2a). The along-track error accounts for the tangential component of the position error expressed originally in a global coordinate system, while the off-track error accounts for the position error that is orthogonal to the vehicle’s path—i.e., orthogonal to the true orientation of the moving vehicle.

In principle, in order to compute along-track and off-track precision measures an estimate of the running vehicle steerage (azimuth) is required to convert global precision errors from a geographic coordinate system to the body frame. In this study, however, smartphone devices report precision estimates simply as a scalar value, which correspond to a circular uncertainty metric, and therefore, the along- and off-track precision errors are considered to be of the same size. In contrast, in order to evaluate the trueness of a navigation solution the position errors are calculated against to a reference trajectory which represents the ground truth. In this case, the along- and off-track accuracy of the observed travel path reflects its deviation from ground truth realized by the scalars *d* and *k*, as shown in Figure 2b. Specifically, along-track error represents the error in the direction of movement between the calculated position and the reference trajectory, while the off-track error expresses the lateral offset of the calculated position from the reference trajectory. In particular, in order to ensure that smartphone trajectories are properly assigned to ground truth, the recorded reference trajectory captured originally at a sampling rate of 5 Hz was interpolated at 25 Hz and used for matching the smartphone data collected at 1 Hz. Then, assuming a linear transition model between successive positions in time, the along- and off-track position trueness is computed in a sequential manner following the analysis steps detailed in [40].

Regarding the smartphone IMU sensors, their performance evaluation follows the same methodology as the one followed for position accuracy. Therefore, the originally generated smartphone MEMS acceleration and gyro values are decomposed into their along- and off-track components to adhere vehicle dynamics. Similarly, the IMU observations obtained using the tactical-grade navigation system, are converted to their components in the body frame system, and finally, the differences between the two estimates (i.e., positioning trueness) are processed and visualized. Notably, in this study, particular attention is paid on the along- and off-track acceleration values as well as the up-track (i.e., vertical) gyro, considering that these measures reflect the dominant motion characteristics of the vehicle.

## 4. Navigation Sensors, Driving Scenarios and Field Work

### 4.1. Tested Smartphones and High-End Navigation System

Two contemporary smartphone devices are tested in this study, particularly the HTC One S and the iPhone 5s. Although these devices are not the latest models released by their respective manufacturers, they are still very recent ones and extensively used worldwide. Both devices comprise a GNSS receiver and a MEMS IMU system that features three-axial accelerometers and gyroscope sensors. Besides, their processing power enables their operation in seamless mode minimizing the effect of errors induced by components other than the assessed sensors. The Application Programming Interface (API) of the HTC One and iPhone 5s devices are the “Location Manager and Sensor Manager of Android API” and the “iOS Framework” respectively. Table 1 summarizes the technical characteristics of both devices. Notably, the operating systems of the selected devices (Android and iOS, respectively), if combined, they represent more than 95% of the smartphone market share for 2015 [41], indicating their broad worldwide use.

The navigation system used to provide the reference trajectory during the tests is the integrated GNSS/IMU SPAN^®^ system produced by NovAtel^®^ (Table 2). The system features a dual frequency (L1/L2) GNSS receiver (NovAtel ProPak-V3) and a high-end, tactical-grade IMU system (iMAR IMU-FSAS). Data processing involved filtering the navigational data using an advanced Kalman filter algorithm (NovAtel^®^ Inertial Explorer) to produce the optimum blended navigation solution that minimizes the effects from GNSS signal multipath and shadows.

### 4.2. Vehicle Cruising and Maneuvering Tests

As already stated, the focus in this study is to test the potential and limitations of smartphone localization capabilities for transport positioning and tracking applications. Contrary to previous studies, our interest focuses on vehicle trajectory extraction and attitude determination. The testing scenarios include two series of field tests. Cruising tests are undertaken to study smartphone performance during normal driving conditions in urban environment, including single and dual carriage way road segments with and without environmental obstructions, such as high-rise buildings and trees. The second set of trials features a number of maneuvering events undertaken at an area with direct reception of satellite signal. These tests involve standard speeding, stopping, turning and other maneuvering events aiming at studying smartphone performance during abrupt changes in their kinematic characteristics and particularly in vehicle attitude. Table 3 summarizes the complete test series.

### 4.3. Sensor Setup and Field Experiments

**Figure 3:**
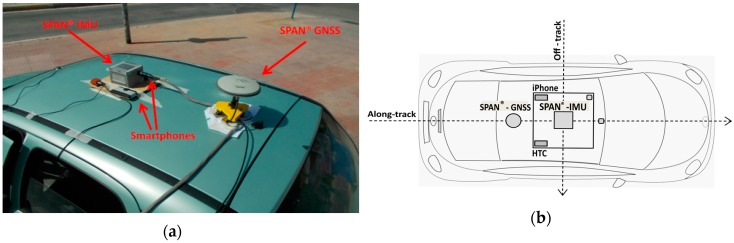
Navigation sensors mounted on top of the test vehicle.

Field testing employed a small passenger vehicle. Specifically, the geodetic-grade IMU unit and the smartphone devices were attached firmly, close to the centre of the test vehicle rooftop using an on-purpose built platform, while the GNSS antenna was fixed in the longitudinal vehicle axis as shown in Figure 3. Also, care was taken so that the IMU unit and both smartphones were aligned to the body frame of the vehicle. Smartphones were firmly attached on the platform using Velcro type fabric hook and loop fasteners. Finally, prior to data collection a dimensional survey was undertaken to compute the relative locations among all sensors. Particularly, the lever-arms between sensors were measured with accuracy better than 0.5 cm. The navigation solution obtained using the NovAtel^®^ Inertial Explorer software and corresponds to the IMU origin.

The cruising tests involve driving the test vehicle along a 4.5 km long trajectory nearby the city centre of Athens, Greece. The road environment involves a dual carriageway part (at start/end), and a deeply urban part with single carriage and narrow streets. The maneuvering tests were performed at an open area within the parking facilities of the former airport of the city of Athens in the Ellinikon area.

## 5. Reference Trajectory Establishment and Smartphone Data Pre-Processing

### 5.1. Reference Trajectory Establishment

In land vehicle navigation applications, several processing strategies are possible for estimating the reference trajectory. Depending on integration level, the loosely coupled GNSS/INS and tightly coupled GNSS/INS techniques may be used. Both approaches result in a continuous trajectory but still they require differential GNSS corrections made available from a base station. Howbeit, they differ in the sense that loose coupling refers to fusing GNSS positions with IMU data, while the tight coupled solution directly combines raw GNSS data (pseudoranges and Doppler) with the inertial information [42]. In principle, the loose coupling of Post-Processed Kinematic (PPK) GNSS data may result in a better solution compared to the tightly coupled one, provided that the quality of the PPK GNSS solution is high enough [42]. Here, we use the loosely coupled approach as the PPK GNSS solution is overly of adequate quality. Besides, as a means of independent check for verifying and improving further the reference trajectory we used a high accuracy orthophoto map of the area for which a number of check points reveal an accuracy better than 0.3 m.

#### 5.1.1. Vehicle Cruising Tests

**Figure 4:**
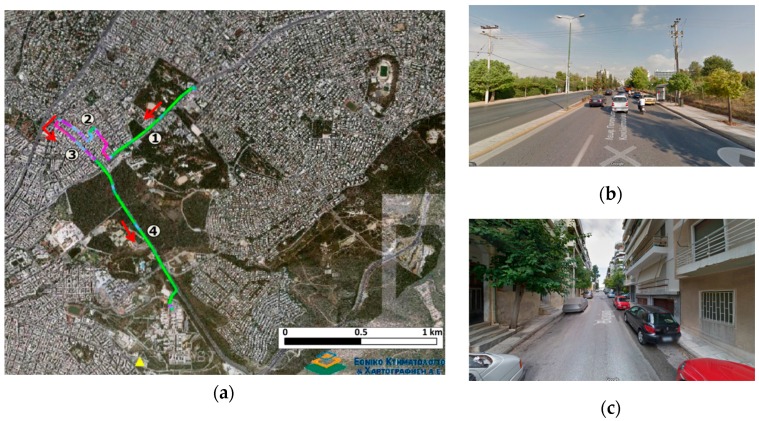
Reference trajectory of cruising scenario (**a**), open-sky (**b**), and obstructed sky conditions (**c**).

This test run was designed to simulate a vehicle trajectory commonly found when driving in urban environments. As shown in Figure 4a, it consists of four sections from which, Section 1 and Section 4 include a dual carriageway road with open sky conditions for most of the time. Section 2 represents a thoroughly urban environment with narrow streets and high-rise buildings. Finally, Section 3 refers to a road segment with tall trees of relatively dense foliage rising along both sides of the road, resulting in severe attenuation and partial blockage of the satellite signal.

**Figure 5:**
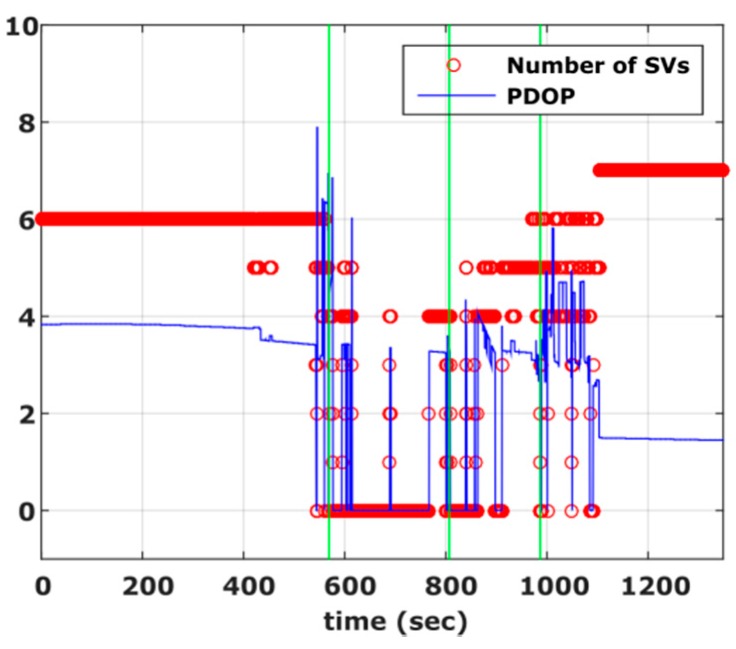
Cruising scenario. Number of visible satellites and PDOP values obtained for the complete travel path. The vertical lines represent the start/end of the deep urban and tree covered sections respectively.

**Figure 6:**
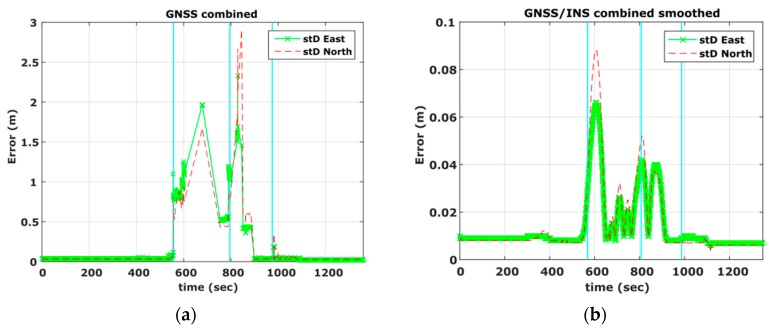
Estimated standard deviation for the GPS-only navigation solution (**a**), and for the smoothed/combined GPS/IMU navigation solution (**b**).

Figure 5 shows the Position Dilution of Precision (PDOP) values and number of satellites visible to the rover receiver and clearly demonstrates the environmental conditions discussed for Section 1, Section 2, Section 3 and Section 4. Specifically, it is evident that PDOP values rise drastically in segments of low or complete loss of satellite visibility (600 s–1100 s). As shown in Figure 6a, these operating conditions result in a poor navigation solution for the PPK GNSS-only case. Particularly, a standard deviation error of 1 m to 2 m is obtained, while there are smaller sections that no position solution is possible to obtain. In an attempt to improve the quality of the reference trajectory we produced the tightly coupled solution, which however didn’t improve the final results due to the high drift in the IMU observables for the periods missing GNSS observables. Finally the reference trajectory adopted in this study was produced employing the loosely coupling fusion technique, augmented by a number of control points acting as constraints in the filter solution, resulting in maximum standard deviation in the horizontal positions of the order of 0.09 m (Figure 6b). The control points were marked by the road intersections at segment 2 and 3 that correspond to adverse signal reception conditions and surveyed prior to field testing using conventional geodetic techniques. Time tagging of the vehicle passing over the control points was feasible due to relatively low (<30 km/h) driving speed. The reference trajectory, shown in Figure 4 is colored to indicate position quality ranging from worst (red) to excellent (green).

#### 5.1.2. Vehicle Maneuvering Tests

**Figure 7:**
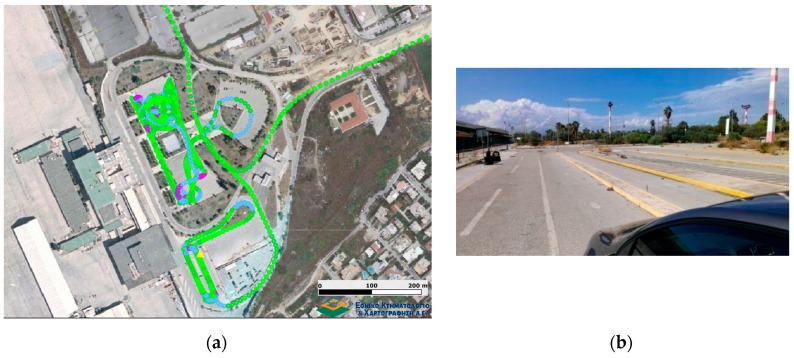
Reference trajectory of vehicle maneuvering scenario (**a**), and testing area snapshot (**b**).

**Figure 8:**
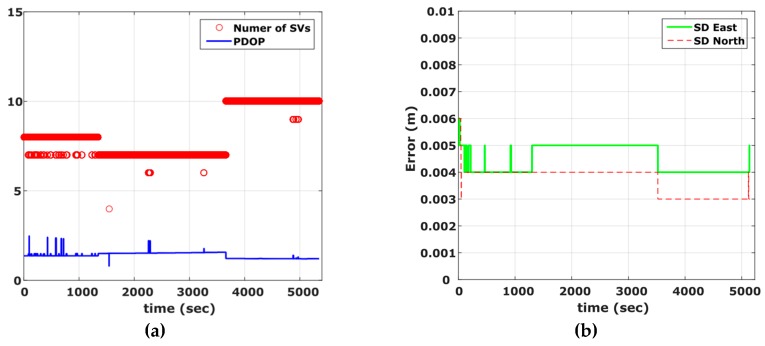
Number of visible satellites and PDOP values for the vehicle maneuvering test (**a**), and estimated position standard deviation (**b**).

Contrary to the previous experiment, the open sky conditions (Figure 7) incurred during the maneuvering trials have resulted in a high quality navigation solution. As shown in Figure 8, PDOP values never exceed a value of 3 while the number of visible satellites was between seven and 10. The final trajectory solution was produced using a loosely coupled fusion scheme, featuring sections of zero velocity (ZUPT), and two-way smoothing, resulting in very low (<0.01 m) standard deviation values in the final horizontal positions. Particularly, the ZUPT function allows the automatic detection of stationary periods, which assist in controlling velocity errors resulting from drift in the IMU observations and thus, improving the final accuracy of the navigation solution.

### 5.2. Smartphone Data Pre-Processing

Smartphone data acquisition was performed using third-party software named SensorLog [43,44], that is, a mobile app available for both iOS and Android devices. SensorLog records raw sensor data and generates ASCII files that include the GNSS position and IMU (accelerometer and gyroscope) observations.

The first stage of smartphone data pre-processing includes parsing the observable files by sensor type. Data recordings are then re-sampled to the desired sampling rate and are synchronized. Notably, the foregoing process is necessary as it allows direct comparison between smartphones operating in different sampling rates. In our case, originally, the SensorLog reads the iPhone 5s data at a sampling rate of 100 Hz both for the GNSS (densified values interpolated from 1 Hz) and IMU sensors, while for the HTC One S generates GPS and IMU data 1 Hz and ~65 Hz, respectively. Thereby, the data sampling rate used for subsequent analysis is 1 Hz for GNSS positions and 10 Hz for the IMU readings.

## 6. Smartphone Data Analysis and Evaluation

### 6.1. Cruising Data

**Figure 9:**
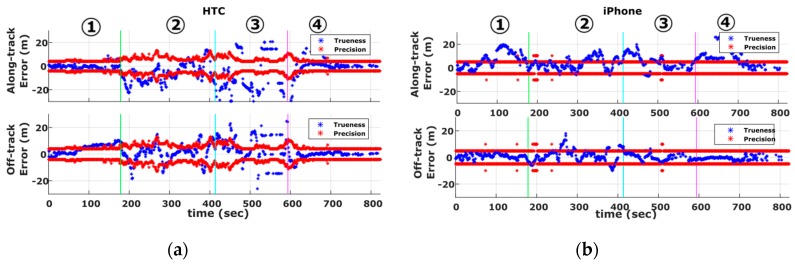
Along-track and off-track GNSS trueness and precision measures obtained for the HTC One S (**a**) and iPhone 5s (**b**) during the cruising scenario.

Smartphone cruising tests aim at estimating vehicle trajectory in absolute terms, and therefore, our main focus is placed on GNSS receiver performance assessment. Figure 9 shows the positioning precision and trueness time series obtained for the complete vehicle trajectory in the along- and off-track directions. From these plots a number of points are evident. The first thing to note is that the along-track error (i.e., trueness) obtained for both smartphones (shown in blue) exhibit larger values compared to off-track trueness (also shown in blue). Moreover, the along-track trueness exceeds the precision estimates (shown in red) for a significant part of the traveled trajectory. This finding indicates the inefficiency of smartphones to produce realistic precision values in the along-track direction for part of the trajectory. In contrast, as expected, off-track trueness (shown in blue) lies within the boundary zone defined by the smartphone precision estimates.

A closer examination of Figure 9 reveals that the accuracy values produced for iPhone 5s are more stable compared to those obtained for the HTC One S, suggesting a somewhat different approach in raw GNSS data treatment by the different manufacturers. Particularly, it appears that the underlying data filtering technique used for the HTC One S, allows the navigation solution to adapt rapidly to environmental conditions. As a result, precision and trueness increase considerably in Section 2 and Section 3 due to adverse satellite signal reception conditions. Contrarily, the iPhone 5s solution appears to be more conservative to external effects, which make it look less realistic, especially for the precision estimates. On the other hand, the final solution of iPhone 5s is affected less by gross errors. Finally, as already discussed, we observe some relatively higher error estimates in the along-track direction compared to those observed in the off-track direction. In fact, these results are in contradiction to the behavior commonly found in urban canyons as satellite geometry tends to be stronger in the along-track direction [45,46]. The findings observed in this case might be due the higher vehicle dynamics in the direction of movement as a function of the processing (filtering) technique that smartphone manufacturers may apply in the GPS data; however, the actual reason for this phenomenon remains unclear.

**Figure 10:**
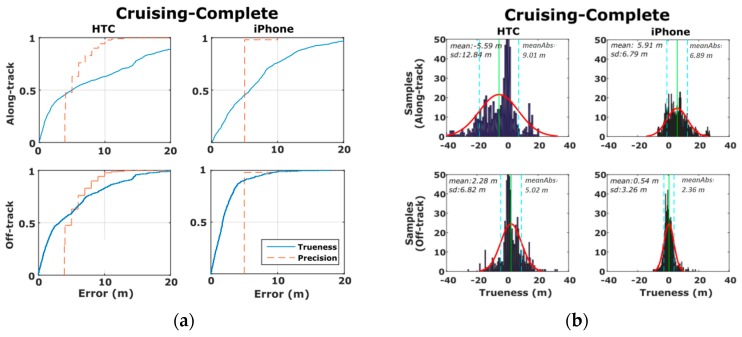
CDF-Cumulative distribution functions (**a**), and probability density histograms (**b**) of the GNSS trueness and precision measures obtained for the complete run of the cruising tests.

Figure 10 shows the previously described accuracy estimates using the cumulative distribution and probability density functions. Clearly, more than 60 and 80% of the positioning trueness derived for the HTC One S and iPhone 5s off-track estimates respectively, lie within the boundary zone defined by their predicted precision values. In contrast, these figures drop down to about 50% for the along-track estimates. Moreover, the reported accuracy values for the iPhone 5s appear to be overly smaller compared to those for HTC One S. Specifically, the error probability density plots obtained for HTC One S reveal relatively higher and more dispersed values departing from their normal distribution bell fit (see Figure 9b).

**Figure 11:**
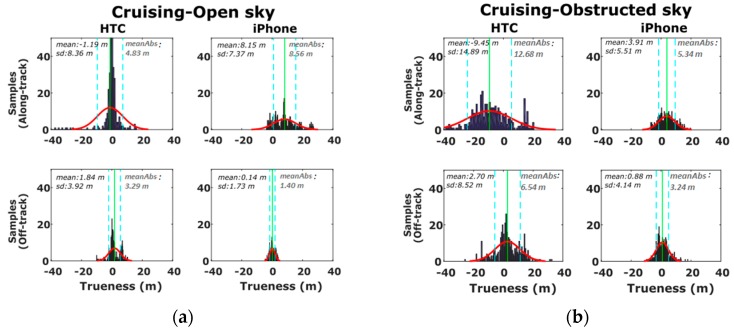
Probability density histograms of the along- and off-track smartphone GNSS trueness for the open-sky (**a**), and obstructed-sky (**b**) sections of the cruising scenario.

In order to examine the effects of the operating environment on positioning errors, we reconstruct the probability densities of accuracy estimates separately for the open sky (Section 1 and Section 4) and obstructed sky (Section 2 and Section 3) segments as shown in Figure 11. From these plots is directly evident the deterioration in the mean and standard deviation errors estimates for Section 2 and Section 3 for which the vehicle travels at partially obstructed signal reception conditions (Figure 11b). Moreover, note that Section 2 and Section 3 feature high-rise (~20 m) buildings and leafy trees rising at both sides of the road, whereas, due to low driving speeds, ranging from 20 km/h to 45 km/h, a time span of about 400 s is required to travel these sections. Contrarily, the significantly lower error values observed in the open sky sections (Figure 11a) reveal the high capabilities and the potential of smartphone GNSS positioning in open areas.

### 6.2. Maneuvering Data

Contrary to vehicle cruising tests, maneuver recognition testing focuses on IMU sensors performance evaluation due to their ability to describe better the variations in motion dynamics. Therefore, maneuver recognition tests take place in open sky conditions, while the assessment of GNSS performance is of lesser importance, also due to its low sampling rate (1 Hz) that makes hard to describe efficiently vehicle dynamics.

#### 6.2.1. Speeding-Stopping Maneuvers

This set of trials aims at studying the sensitivity and accuracy of smartphone x-acc (along-track) acceleration values to describe the vehicle dynamics while it performs a speeding/stopping maneuver. Also, the y-acc (off-track) acceleration and z-gyro (azimuth) are concerned, as they provide side information regarding the lateral control of vehicle dynamics.

**Figure 12:**
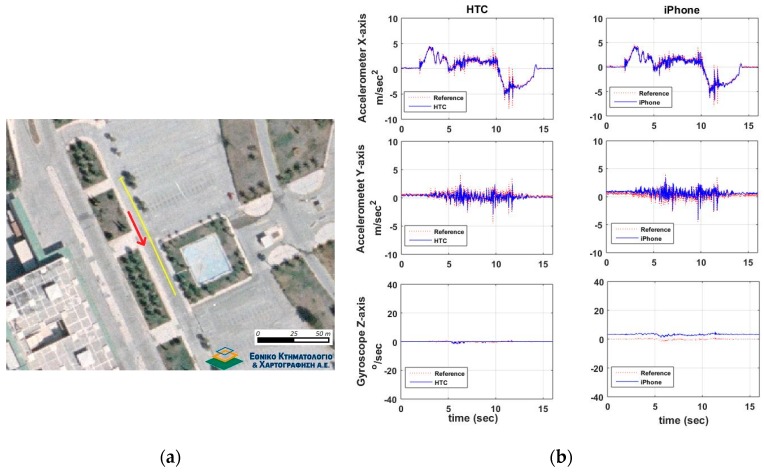
A typical speeding-stopping maneuver that consists of an aggressive acceleration and deceleration part on a straight line path. Travelled trajectory (**a**) and smartphone IMU sensor readings (**b**).

**Figure 13:**
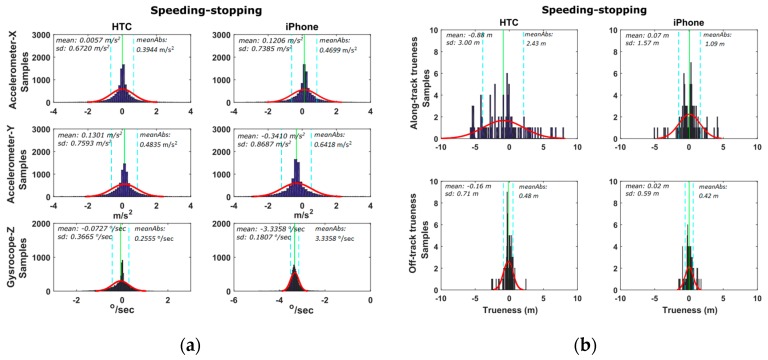
Probability density distributions obtained for the smartphone trueness for the IMU (**a**), and GNSS (**b**) readings considering the complete set of the speeding-stopping tests.

Figure 12 shows a typical speeding/stopping maneuver example, as well as, the time series differences for x-acc, y-acc and z-gyro computed between the smartphone readings and those obtained from the high-end GNSS/IMU system. An extremely good match between all three systems is evident, that indicates the high capabilities of smartphone IMUs. Similarly to vehicle cruising tests, we produce the probability density distributions for the differences obtained between the smartphone and the reference navigation system estimates. These estimates use the data for all six speeding/stopping tests described in Table 2. Figure 13 shows the respective distributions for the IMU (x-acc, y-acc, z-gyro) and the GNSS estimates. Notably, the results drawn in these plots rely on the complete set of trials undertaken for each maneuver type shown in Table 3. All plots obtained for the IMU sensors reveal a good agreement between smartphone recorded kinematics and the reference values, following a nice pattern not departing significantly from the normal distribution shape. Notably, the standard deviations obtained for x-acc are very small for both smartphones, while HTC One S exhibits a better mean value. The probability distributions for the GNSS errors are similar to those obtained for the cruising tests, indicating also the low sampling rate that make them less favorable for dynamic applications.

#### 6.2.2. Turning Maneuvers

**Figure 14:**
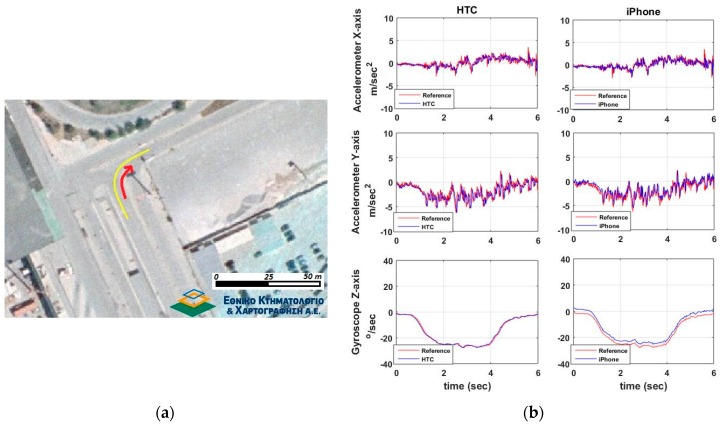
A typical turning maneuver of a 90° right turn. Travelled trajectory (**a**), and smartphone IMU sensors readings (**b**).

**Figure 15:**
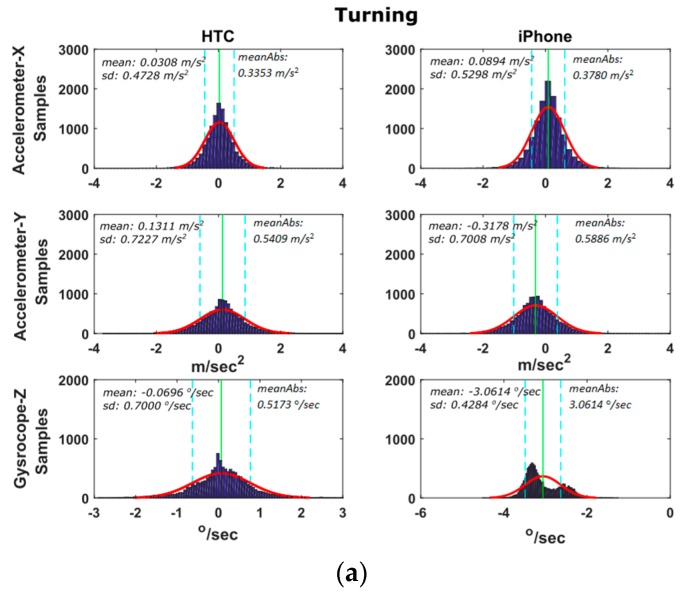

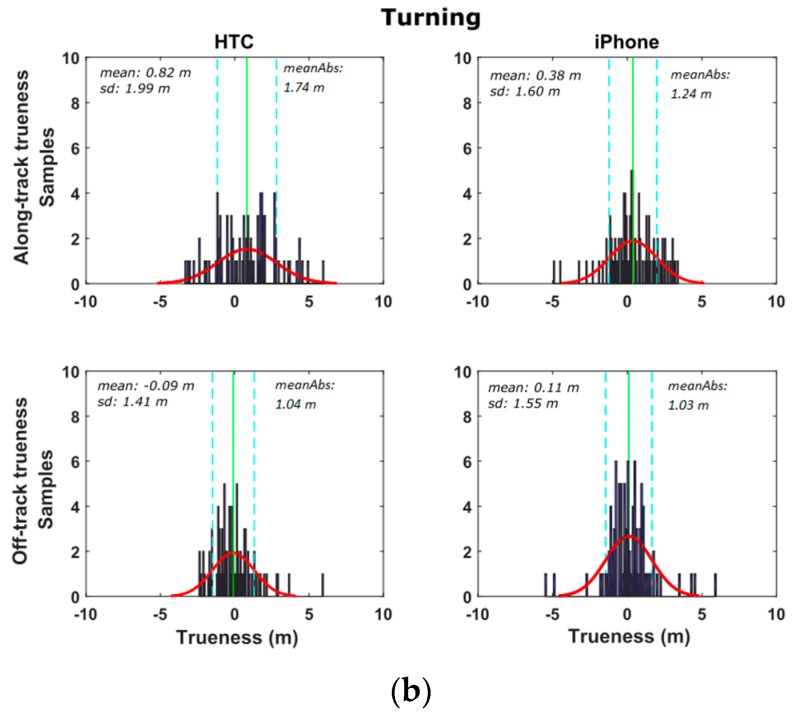
Probability density distributions obtained for the smartphone trueness for the IMU (**a**), and GNSS (**b**) readings considering the complete set of the turning tests.

Here the focus is again on the x-acc, y-acc and z-gyro estimates recorded as the vehicle performs a series of left and right turning events. However, the emphasis is placed on y-acc given that lateral accelerations are of more importance while turning. Figure 14 shows an example of a right turn at 20 km/h driving speed and the corresponding sensor differences. Similarly to previous tests, Figure 15 shows the distributions of the differences between smartphone and high-end navigation system estimates for all fourteen turning maneuvers shown in Table 2. Examination of the y-acc values show that the HTC One S exhibits a lower (by a factor of 2) mean value difference compared to the one derived for the iPhone 5s, while both smartphones result in similar standard deviations. A closer examination of Figure 15 reveals an interesting observation regarding z-gyro differences for the iPhone 5s device. The double-bell shaped distribution obtained for z-gyro reveals a somewhat uncommon behavior; however, its origins remain unclear.

#### 6.2.3. Special Maneuvers

**Figure 16:**
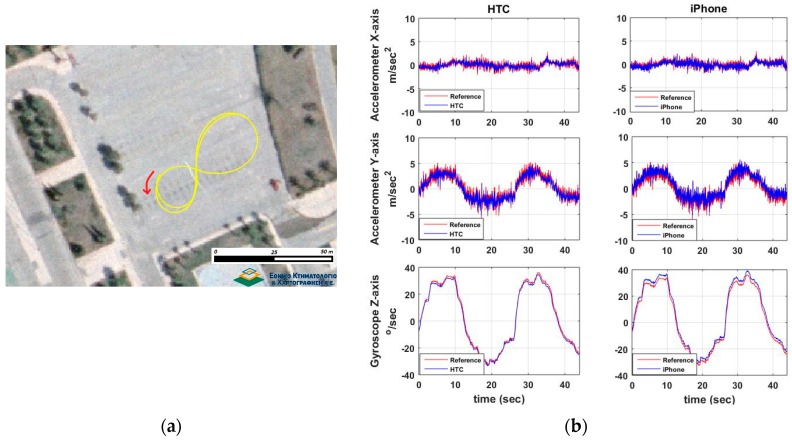
The Figure 8-shaped special maneuver. Travelled trajectory (**a**), and smartphone IMU sensor readings (**b**).

**Figure 17:**
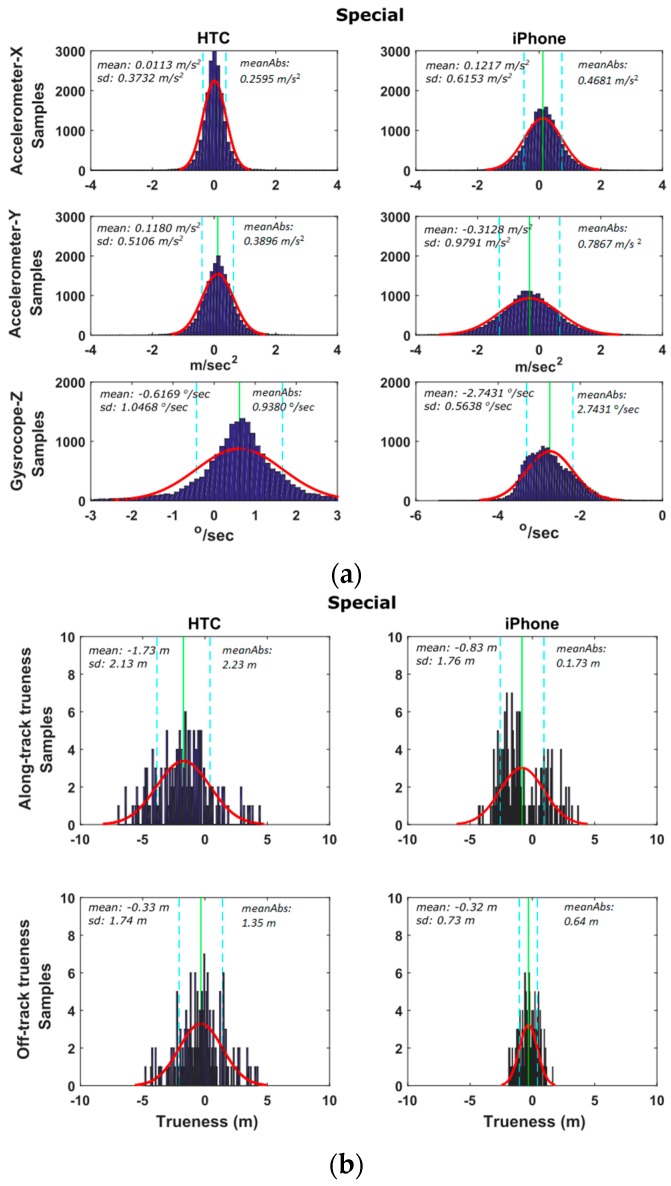
Probability density distributions obtained for the smartphone trueness for the IMU (**a**), and GNSS (**b**) readings considering the complete set of special tests.

This last series of trials involves a number of less standardized maneuvering events usually performed at emergency conditions or maneuvering events of a more complex character. As an example, Figure 16 shows the results obtained for a Figure 8-shaped maneuver performed at ~25 km/h driving speed. The agreement between smartphone recordings and high-end GNSS/IMU system is apparent despite the highly noisy acceleration values, which are particularly evident for the y-acc component. The fine resolution of z-gyro observations is also apparent. Note that in these diagrams, the IMU observations obtained for the reference trajectory appear to be nosier compared to those derived for the smartphones due to their significantly higher sampling rate. Figure 17 shows the distributions of the differences between smartphone and high-end navigation system estimates for the special maneuvers shown in Table 3.

## 7. The Role of Smartphones as a Driver for ITS Services

As expected, the foregoing test analysis show remarkably that the actual capabilities of contemporary smartphones to capture reliably a vehicle trajectory can vary, and depend on numerous factors, among which the operating environment is probably the most dominant. Therefore, from a user perspective and at an application level, the question then arises: What are the limitations in smartphone positioning and what are their implications for ITS services?

**Figure 18:**
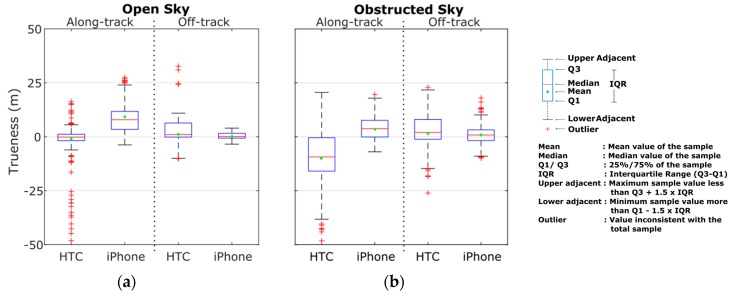
Box and whiskers plot obtained for the GNSS trueness for the open sky (**a**), and obstructed sky conditions (**b**) considering the complete test series of the vehicle cruising trials.

The answer to this question lies on user specific requirements and threshold values imposed by application type. Figure 18 summarizes the statistics for smartphone GNSS position accuracy (trueness) obtained considering the complete datasets collected in this study. Summary results are depicted in a box-and-whisker diagram [47], so that the complete set of data quartiles as well as the key indicators used to define dispersion, skeweness and outliers in the data are evident in a single plot. Therefore, direct comparison of positioning performances between different operating environments and smartphone devices is possible. As an example, the results obtained for the open sky conditions and the along-track solution, reveals a shift in the error distributions between the HTC One S and the iPhone 5s with the latter exhibiting much higher errors. Cross comparisons of these findings with the error histograms shown at the top of Figure 11a verify these results. Moreover, the 25th percentile for the iPhone 5s is approximately 5 m, which is slightly larger to the 75th percentile for the HTC One S. Specifically, 25% of errors for the iPhone 5s is of the order of 5 m or less as compared to 75% for the HTC One S. Also, there are many outliers at both the high and low ends of the distribution for HTC One S, while there are less outliers at the high end of the distribution for the iPhone 5s. Obviously, more data collected at different environments are required to fully characterize sensor performances; howbeit, the results presented here are considered to be representative.

Regarding the IMU sensor performances, it can be said that considering their extremely low price, they are well perceived in contemporary mobile phones and they perform substantially well in numerous applications, despite a number of spotted deficiencies; for instance, those identified in this study (e.g., shape of z-gyro distribution shown in Figure 15) and elsewhere in the social media, such as free blog hosting services. In the last five years, the improvement in mobile phone IMU chipsets has been remarkable, indicating their high potential for massive user ITS applications.

**Figure 19:**
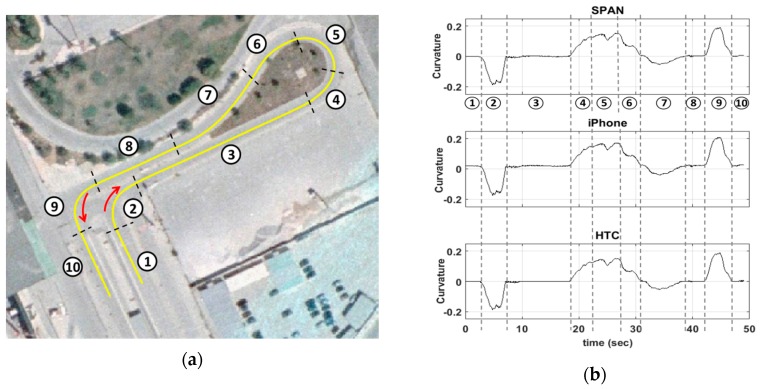
Road curvature compution using smartphone IMUs. Travelled trajectory (**a**), and extracted road curvature diagrams (**b**).

**Figure 20:**
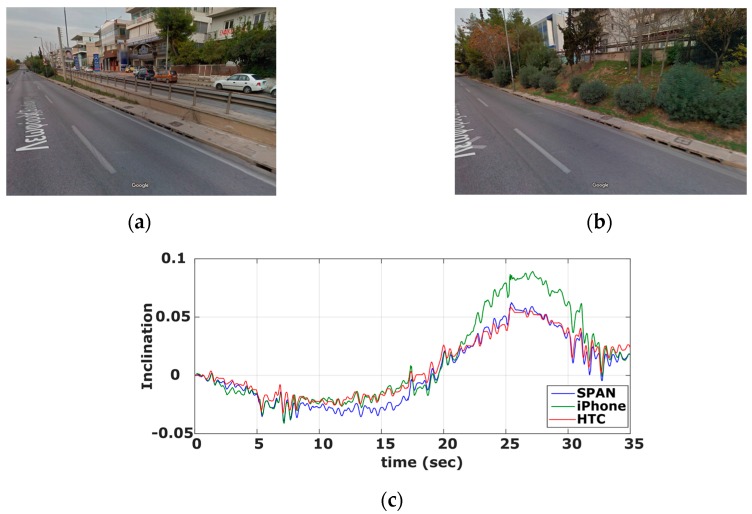
Vertical profile of exit ramp computed using smartphone gyroscopes. Ramp elevation at entrance (**a**) and exit points (**b**), and computed road inclination (**c**).

Notwithstanding a great number of ITS services relies directly on the navigation solution produced using a positioning terminal (e.g., location, velocity), a great number of applications asks for supplementary information, which can be generated based on raw positioning data. Computing road geometry characteristics, such as the curvature of centerline alignment and longitudinal/lateral inclination are typical examples for many road applications. For instance, road curvature forms a standard product for inventory surveys, which is currently produced using sophisticated navigation systems as part of an integrated Mobile Mapping System (MMS) [37,48]. However, road curvature can be extracted in a sequential manner, based on azimuth differences estimates produced from subsequent sums of z-gyro values; and thus, leading to better accuracy statistics for the road curvature compared to that for raw z-gyro. As an example, Figure 19 shows the curvature diagram produced in this study for a complex maneuver using the high-end GNSS/IMU system and the smartphone devices. The apparent small differences reveal the high potential of low-cost systems. Notably, such observations are of particular importance today, as effort is made in many research centers (for example see [22,49]) to make a shift from expensive MMS to low-cost systems that could be used for services featuring more relaxed quality requirements.Similarly, other information, such as an accurate representation of the profile of a vertical alignment is necessary for fuel estimation models and for eco-driving behavior studies (for example see [50]). Notably, longitudinal inclination of a road alignment might be produced using smartphone IMU pitch (y-gyro) values. To this end, Figure 20 shows the vertical profile computed for an exit ramp of a motorway using the reference navigation system and both smartphones units. Again, the small differences observed between the three solutions show the capabilities of modern smartphone sensors for road applications.

## 8. Conclusions

It has been apparent from the results of the tests that modern smartphones are capable of providing a remarkably good navigation solution for a wide range of road and ITS applications. Current GNSS chipsets proved to be very reliable and consistent in open sky environments, while their performance degrades rapidly (by a factor of two) when moving in satellite signal obstructed areas. Smartphone IMUs, despite their drifting nature, proved to be very sensitive and useful for describing vehicle dynamics and for maneuver recognition. Besides, the benefit of using the raw positioning information for computing new data types, such as road geometry features was highlighted. Moreover, analysis revealed remarkable differences in the error budget between smartphones produced by different manufactures, probably due to the variant processing strategies applied by different companies. It is recognized that the capabilities of smartphone GNSS and IMU sensors can be fully exploited if fused with other sensor data (e.g., cameras) or combined with external information (e.g., digital maps); however, this aspect is out of the scope of this study.

## Figures and Tables

**Table 1:** Technical characteristics of tested smartphones.

	HTC One S	iPhone 5s
**OS**	Android 4.1.2	iOS 7
**Processor**	Qualcomm MSM8290 Dual-core 1.5 GHz Krait	Apple A7 + M7 motion coprocessor Dual-core 1.3 GHz Cyclone
**GNSS receiver**	A-GPS	A-GPS + GLONASS
**Accelerometer**	Panasonic EWTSAFM (3-axial)	Bosch Sensortech BMA220 (3-axial)
**Gyroscope**	Model N/A (3-axial)	STMicroelectronics B329 (3-axial)

**Table 2:** Technical characteristics of geodetic-grade GNSS/IMU.

iMAR IMU				
Gyroscope rate bias	<0.75°/h			
Gyroscope noise	0.1°/√h			
Accelerometer bias	1.0 mg			
Novatel SPAN^®^ (post processing)				
Position	Horizontal (RMS)		Vertical (RMS)	
	0.01 m		0.02 m	
Velocity	Horizontal (RMS)		Vertical (RMS)	
	0.020 m/s		0.010 m/s	
Attitude	Roll (RMS)	Pitch (RMS)		Heading (RMS)
	0.004°	0.004°		0.013°

**Table 3:** Summary of vehicle cruising and maneuvering testing scenarios.

**Vehicle Cruising**	~4500 m	open sky/high-speed (avenues) highly-obstructed sky/low-speed (dense-urban) tree-covered section traffic light stops 90° turns
**Vehicle Maneuvering**	speeding/stopping	**Six maneuvers consisting of:** normal/aggressive acceleration normal/aggressive deceleration
	turning	**Fourteen maneuvers consisting of:** right/left U-turns right/left 90° turns right/left 90° turns + brake
	special	**Seven maneuvers consisting of:** overpassing aggressive avoidance maneuvering Figure 8-shape maneuvering circular routing
